# Correction: Expression of modified FcγRI enables myeloid cells to elicit robust tumor-specific cytotoxicity

**DOI:** 10.7554/eLife.101165

**Published:** 2024-07-02

**Authors:** Leen Farhat-Younis, Manho Na, Amichai Zarfin, Aseel Khateeb, Nadine Santana-Magal, Alon Richter, Amit Gutwillig, Diana Rasoulouniriana, Annette Gleiberman, Lir Beck, Tamar Giger, Avraham Ashkenazi, Adi Barzel, Peleg Rider, Yaron Carmi

**Keywords:** Mouse

Farhat-Younis L, Na M, Zarfin A, Khateeb A, Santana-Magal N, Richter A, Gutwillig A, Rasoulouniriana D, Gleiberman A, Beck L, Giger T, Ashkenazi A, Barzel A, Rider P, Carmi Y. 2024. Expression of modified FcγRI enables myeloid cells to elicit robust tumor-specific cytotoxicity. *eLife* **12**:RP91999. doi: 10.7554/eLife.91999.

Published 17 June 2024

After a careful additional review of the manuscript by Klichinsky et al., we noticed three inaccuracies in our description of their research. Firstly, the authors fused only CD3ζ signaling domain to their scFv, without CD28 signaling domain. In addition, they used adenovirus and not adeno-associated virus to transfect the monocytes. Lastly, they did not claim that the effect they found in vivo was induced by adenovirus transduction. Therefore, we revised the paragraph which addresses this study in the Discussion.

**Corrected text in Discussion:**

Recently, Klichinsky et al. have attempted to genetically modify macrophages using an adenoviral vector Ad5f35 expressing αCD19 and αHER2 Chimeric Antigen Receptor fused to CD3ζ signaling domain. The authors suggested that CAR expression and activation in human macrophages could be employed as a strategy to kill tumor cells expressing the corresponding antigens through phagocytosis (Klichinsky et al., 2020). It may be that the use of Adenovirus as transduction vectors, or human macrophages cultured from blood monocytes enable overcoming the limitations described in the present manuscript.

**Originally published text in Discussion:**

Recently, Klichinsky et al. have also attempted to genetically modify macrophages using an adenoviral vector expressing αCD19 and αHER2 Chimeric Antigen Receptor fused to CD3ζ − CD28 signaling domains. They suggested that this strategy can be applied to kill HER2-expressing tumor cells through phagocytosis. While the authors demonstrated expression by flow cytometry, they did not attempt to identify the membranous localization in macrophages. It may also be that the use of Adeno-associated viruses (AAV) as transduction vectors enable overcoming the inflammatory cascade induced in myeloid cells as a result of liposome-based transfection or lentiviral transduction, thus enabling expression. With that being said, the authors concluded that the improved survival induced in mice was due to M1-like phenotype induced in transduced macrophages by the virus, rather than the CAR construct (Klichinsky et al., 2020).

The article has been corrected accordingly.

